# Transseptal Mitral Valve-in-Valve-in-Valve

**DOI:** 10.1016/j.case.2023.12.031

**Published:** 2024-02-05

**Authors:** Blanaid Canavan, Mark Higgins, Dale J. Murdoch, Christopher Raffel, Katherine Lau, Gregory M. Scalia, Karl Poon

**Affiliations:** a The Prince Charles Hospital, Metro North Health, Brisbane, Australia; b University of Queensland Medical School, Herston, Australia

**Keywords:** Mitral valve, Transcatheter implantation, Valve-in-valve-in-valve, Hemolysis

## Abstract

• Transseptal MV implantation is performed for many indications.
• Previous ViV procedures do not preclude further ViV insertion.
• Appropriate sizing of transseptal MVs is essential to reduce complications.
• Multimodality cardiac imaging is essential for valve placement and follow-up.
• Frequent serial TTE is recommended for follow-up.

## Graphical abstract

**Figure:**
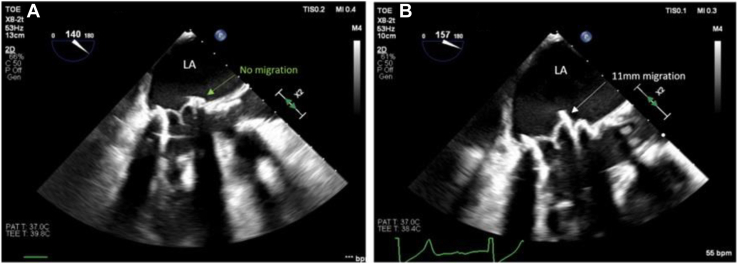

## Introduction

Transcatheter mitral valve-in-valve (ViV) procedures are increasingly used in patients in whom the risk of redo open heart surgery is considered prohibitive.^1^ Rates of immediate and delayed complications are generally low but can occur and pose complex management dilemmas. Multimodality imaging, including transthoracic echocardiography (TTE), transesophageal echocardiography (TEE) and cardiac computed tomography (CCT) can assist in assessing complex cases of valvular regurgitation and facilitate successful transcatheter mitral valve (MV) implantation, even in the presence of a previous MV bioprostheses or ViV prostheses. We present a complex patient with hemolytic anemia who underwent a second mitral ViV procedure because of the unusual complications of delayed partial bioprosthesis dislodgement and valvular hemolysis.

## Case Presentation

An 80-year-old woman presented to the emergency department of a regional hospital with a 6-week history of progressive fatigue, exertional dyspnea, and hematuria. The patient’s medical history included ankylosing spondylitis (controlled on adalimumab and methotrexate), chronic kidney disease, hypertension, hypercholesterolemia, coronary artery disease, and paroxysmal atrial fibrillation. Six years prior, the patient had undergone combined bioprosthetic MV (27 mm) and aortic valve (AV; 19 mm) replacements for degenerative valve disease, with a subsequent redo AV replacement (19 mm) for a degenerating aortic prosthesis 5 years later. One year before the reported presentation, the patient required emergent, out-of-hours transseptal mitral ViV implantation (23 mm), which was performed for torrential, acute mitral regurgitation secondary to flail leaflet leading to acute pulmonary edema and acute decompensated heart failure. Preoperative CCT was not performed, because of patient acuity, and a 23-mm size valve was chosen because of concerns regarding the risk for left ventricular outflow tract obstruction in the presence of the redo AV bioprosthesis.

In the reported presentation, the blood pressure was 135/80 mm Hg, and the heart rate was 65 beats/min. The physical examination revealed a jaundiced, pale complexion accompanied by hepatosplenomegaly. There were dual heart sounds and a new pansystolic murmur on cardiac auscultation. The chest was clear to auscultation in all fields. There was mild pedal edema.

The patient was found to have severe anemia, with hemoglobin of 72 g/L with moderate numbers of red cell fragments (15-20 per 40× high-powered field) and indications of hemolysis (total bilirubin 46 mmol/L, lactate dehydrogenase 2,233 U/L, haptoglobin 0.02 g/L, and reticulocyte count 323 × 10^9^/L). The Coombs test yielded a negative result. Autoimmune screening was also negative. Urine microscopy detected high levels of myoglobin.

A provisional diagnosis of hemolytic anemia was made, secondary to valvular hemolysis. The patient was transferred to our tertiary cardiac center and transfused 1 unit packed red blood cells. TTE was performed and showed a high MV gradient at 9 mm Hg and mild mitral paravalvular regurgitation (PVR). The bioprosthetic AV was well seated, with normal peak velocity (2.7 m/sec), mean gradient (15 mm Hg), and effective orifice area (1.4 cm^2^). There was no aortic regurgitation.

**Figure 1:**
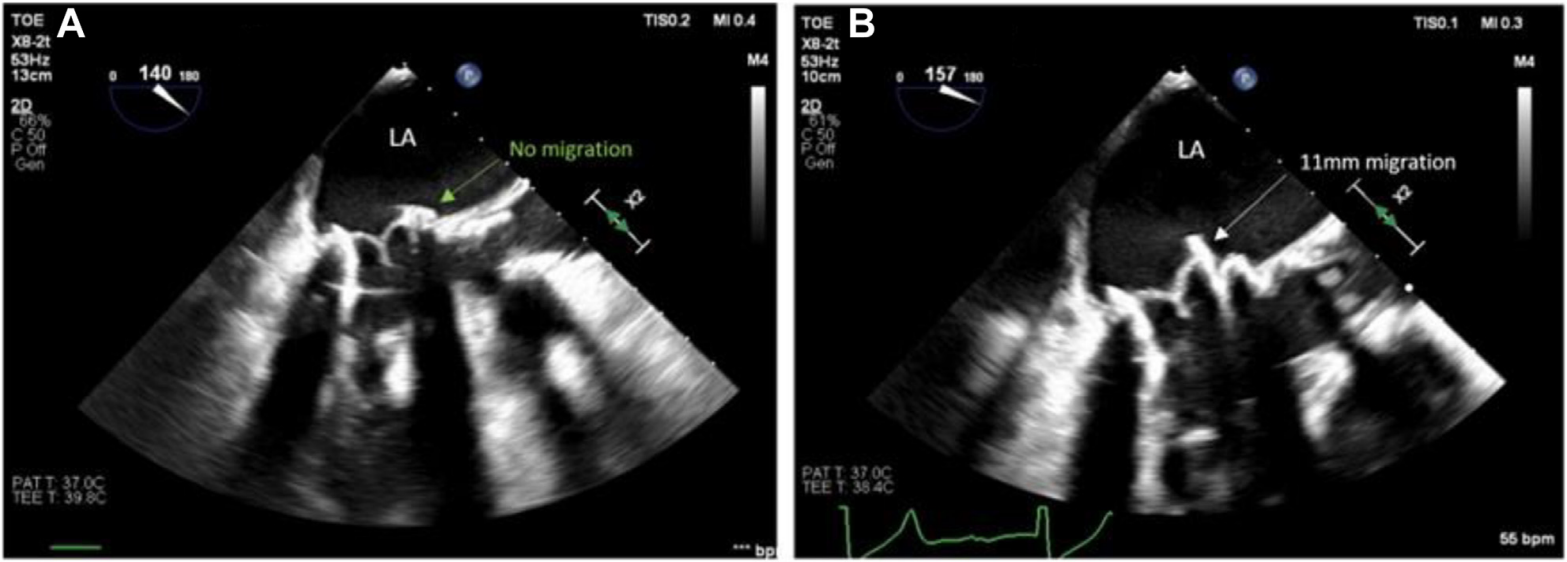
Two-dimensional TEE, midesophageal long-axis (140°) view, systolic phase **(A)**, demonstrates a well-seated mitral ViV (*green arrow*) following the initial procedure and **(B)** long-axis (157°) view, systolic phase, demonstrates migration of the ViV prosthesis (*white arrow*) 11 mm into the left atrium (LA) 1 year later.

**Figure 2:**
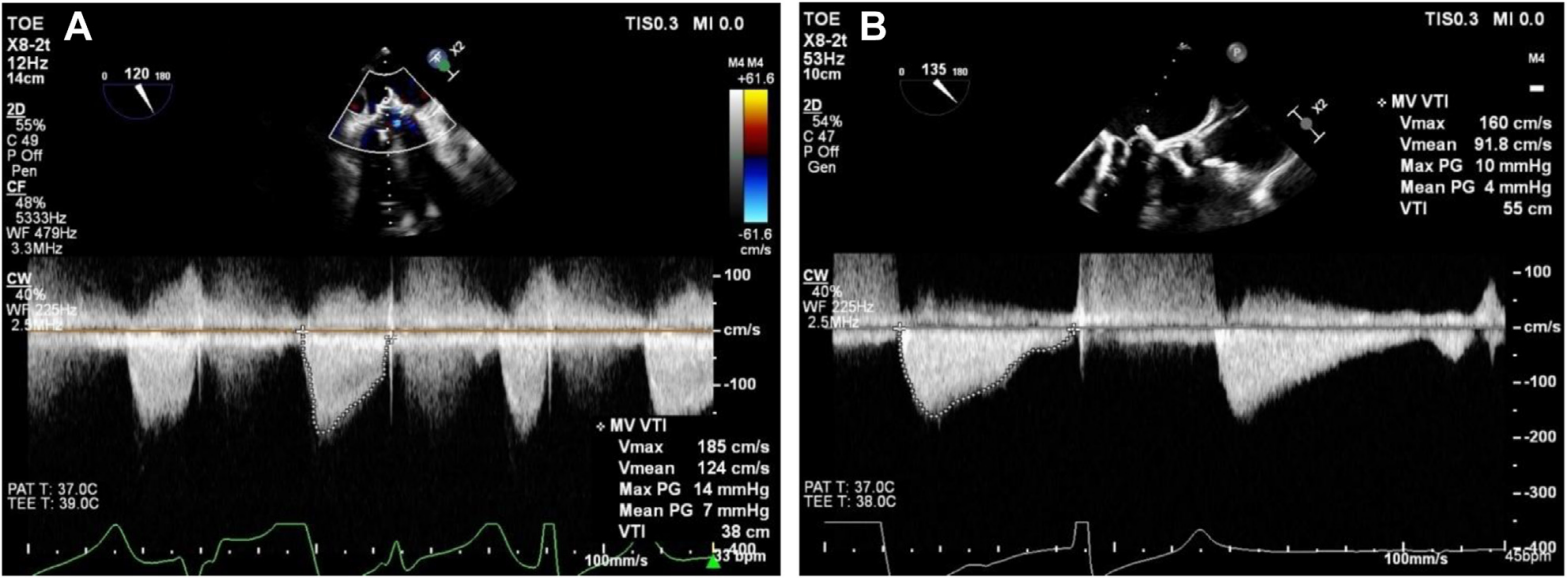
Two-dimensional TEE, midesophageal view with color flow Doppler–guided continuous-wave spectral Doppler display, demonstrates **(A)** high MV mean pressure gradient (7 mm Hg, heart rate 33 beats/min) before mitral ViViV insertion and **(B)** reduced MV mean pressure gradient (4 mm Hg, heart rate 45 beats/min) immediately following mitral ViViV implantation.

TEE demonstrated that the 23-mm transcatheter valve implanted within the 27-mm bioprosthetic MV had migrated into the left atrium (Video 1), particularly the anterior aspect, resulting in significant prosthesis angulation. The valve had partially dislodged at least 11 mm back into the left atrium and appeared at risk for total embolization (Figure 1). The defect measured 6.5 mm in length, forming 10% of the valve circumference. Effective regurgitant orifice area was 0.22 cm^2^. There was severe intervalvular and supraskirtal regurgitation from the lateral to anterior aspect of the MV replacement, between the 9 o’clock and 1 o’clock positions (Videos 2-4). The mean pressure gradient through the valve was 7 mm Hg (Figure 2A). There was no valvular thrombus.

The patient continued to be severely anemic and became transfusion dependent to maintain hemoglobin levels above 80 g/L. The Society of Thoracic Surgeons score was calculated at 21%, and the patient declined third-time open heart surgery. Consensus from the multidisciplinary heart team (interventional and imaging cardiologists, along with cardiothoracic surgeons) was to proceed with a transseptal mitral valve-in-valve-in-valve (ViViV) implantation, aiming to abolish prosthetic PVR by extending the combined new valvular complex further into the left ventricle. CCT was performed to determine valve sizing, confirming valve migration and angulation of the previous ViV prosthesis.

**Figure 3:**
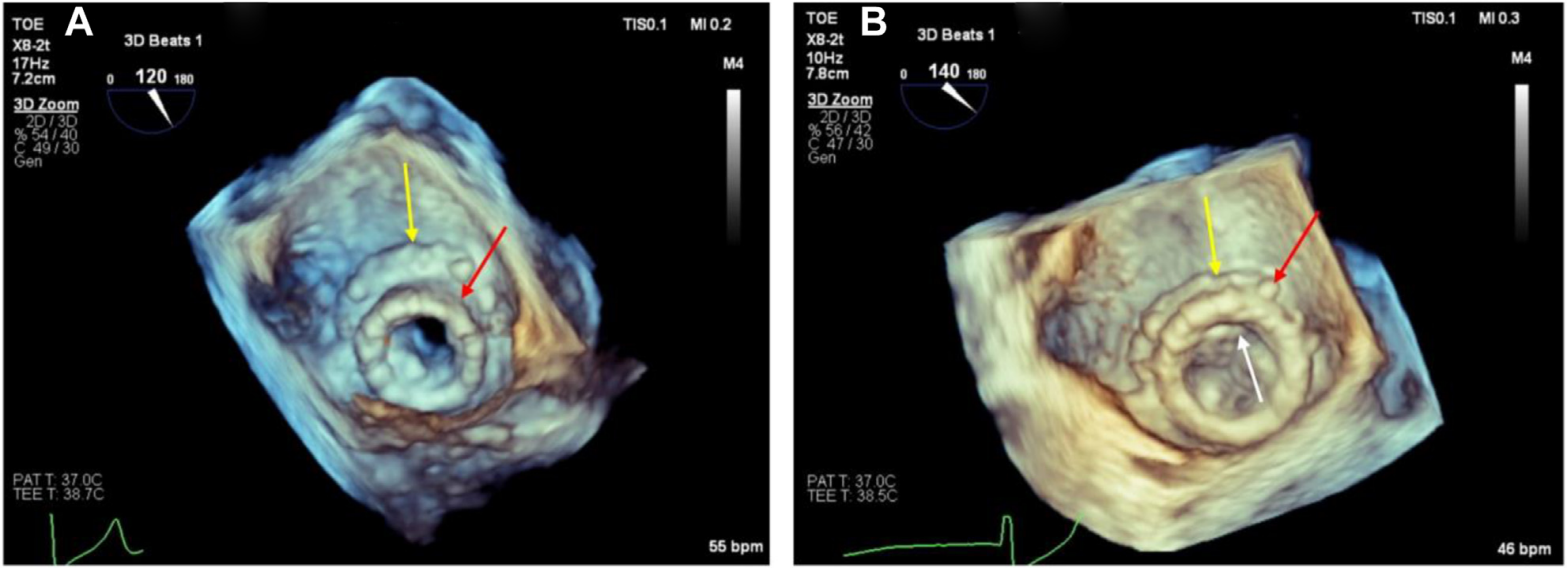
Three-dimensional TEE, volume-rendered reconstruction display, en face view from the perspective of the LA, demonstrates **(A)** the initial 27-mm bioprosthetic MV (*yellow arrow*) and 23-mm ViV (*red arrow*), which has migrated toward the LA, and **(B)** the new 26-mm transcatheter valve implanted during the mitral ViViV procedure (*white arrow*) sitting within the initial ViV prosthesis.

**Figure 4:**
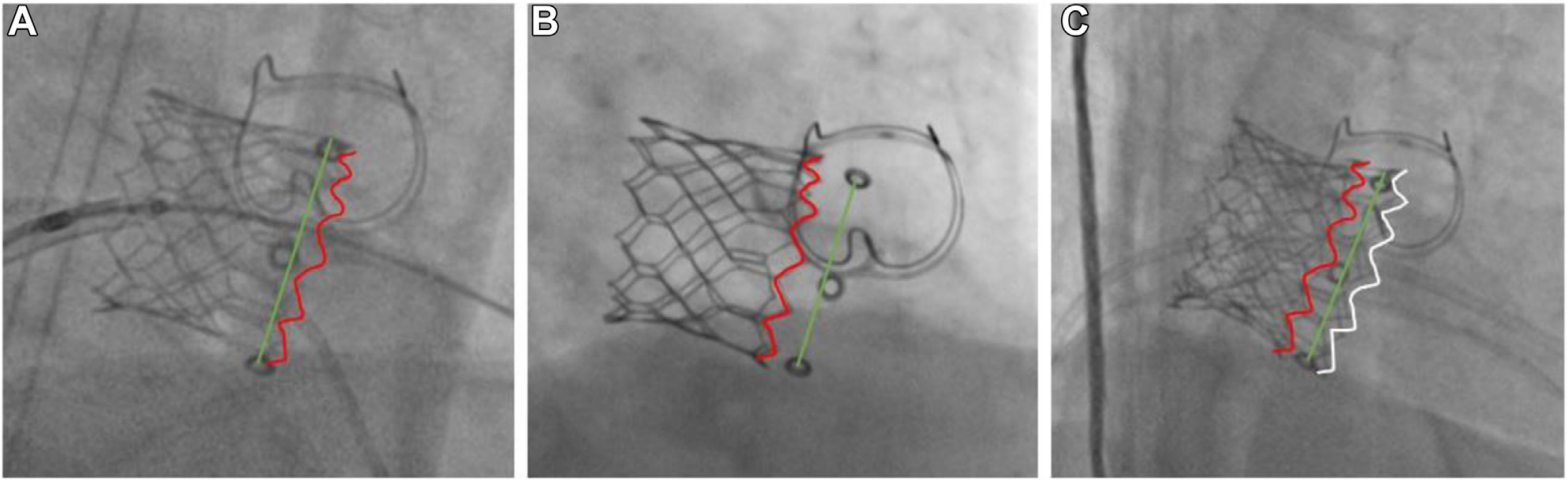
Fluoroscopic images **(A)** immediately following previous ViV procedure, demonstrates 23-mm transcatheter valve edge (*red outline*) in line with islets of previous 27-mm bioprosthetic MV (*green line*). **(B)** After 1 year, migration of 23-mm transcatheter valve (*red outline*) from the islets of the bioprosthetic MV (*green line*). **(C)** Transcatheter 26-mm ViViV (*white outline*) well seated in comparison with bioprosthetic valve islets, preventing further migration of previously placed 23-mm ViV.

The transseptal mitral ViViV procedure was performed under general anesthesia via femoral venous access in a hybrid catheter laboratory and operating theater. A 26-mm valve was positioned within the migrated 23-mm valve under transesophageal echocardiographic and fluoroscopic guidance (Figures 3 and 4).

**Figure 5:**
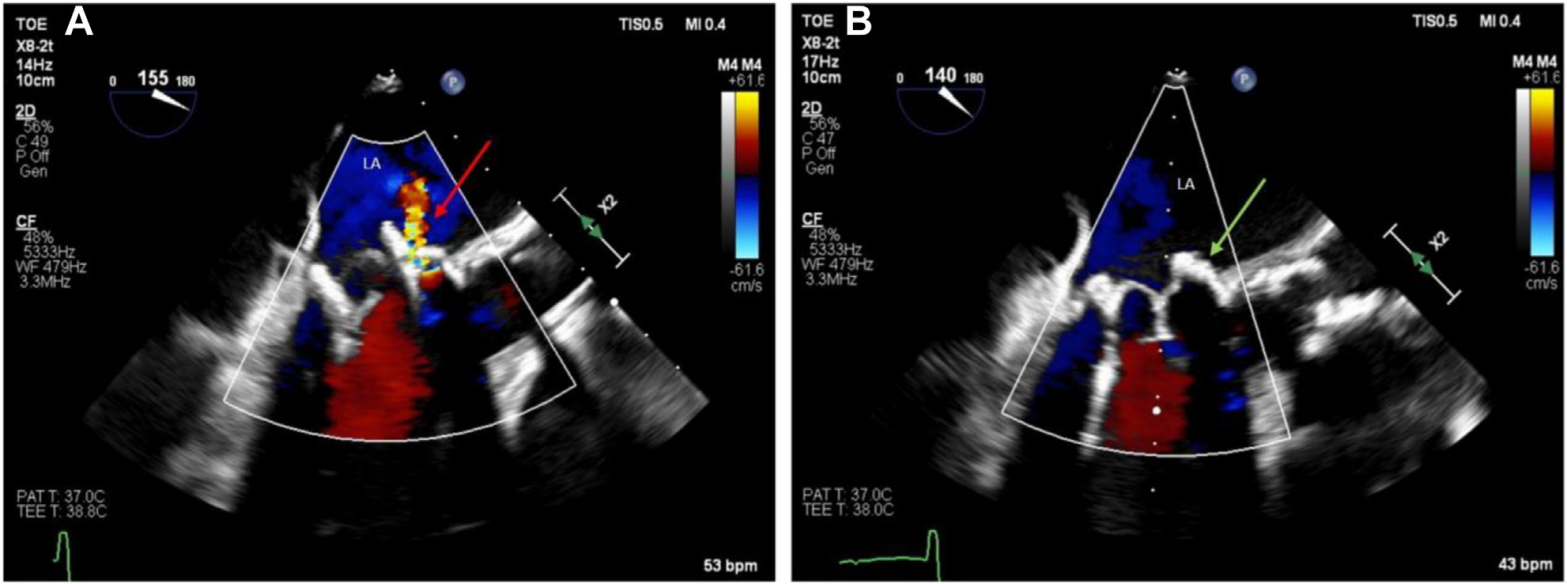
**(A)** Two-dimensional TEE, midesophageal long-axis (155°) view, systolic phase, demonstrates moderate PVR (*red arrow*) between 27-mm bioprosthetic MV and 23-mm ViV, with migration toward the LA. **(B)** Two-dimensional TEE, midesophageal long-axis (140°) view, demonstrates the absence of PVR (*green arrow*) following implantation of the new 26-mm transcatheter valve in previous ViV apparatus.

**Figure 6:**
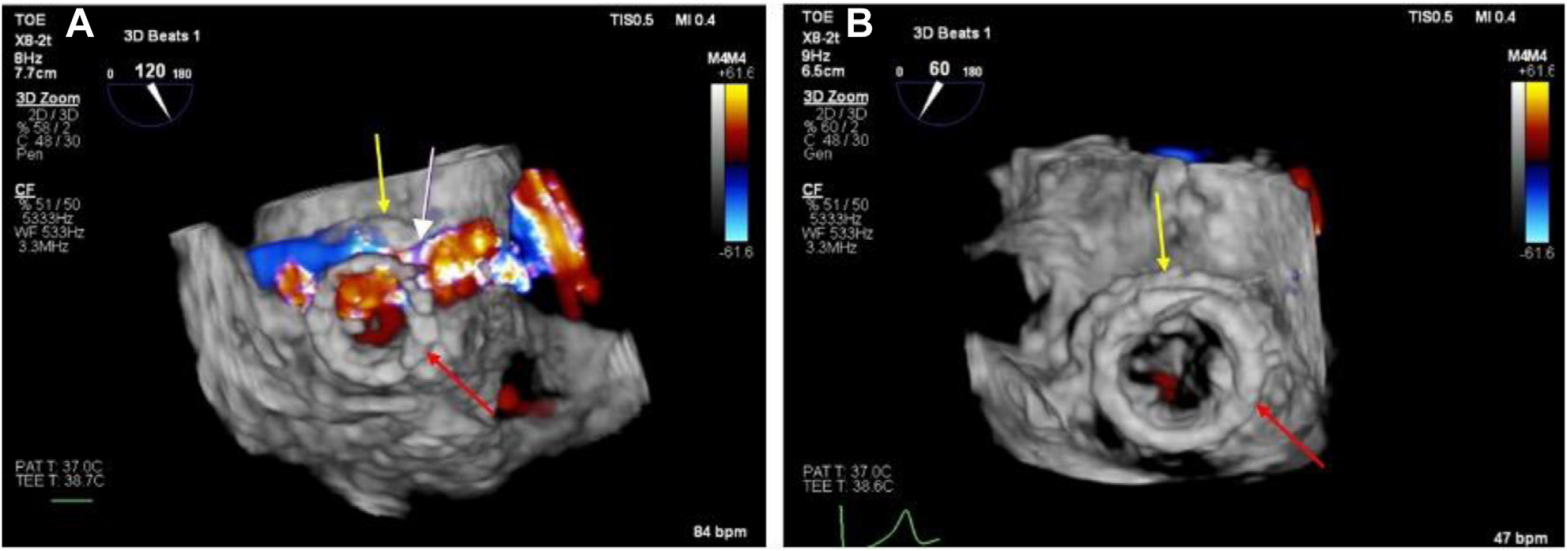
**(A)** Three-dimensional TEE, volume-rendered reconstruction with color flow Doppler display, en face view of the MV from the perspective of the LA, systolic phase, demonstrates paravalvular and supraskirtal regurgitation (*white arrow*) from the 9 o’clock to 1 o’clock positions arising between 27-mm bioprosthetic MV (*yellow arrows*) and 23-mm ViV (*red arrows*) and **(B)** resolution of the PVR following implantation of the new 26-mm ViViV.

Final placement was deemed successful, with the ViViV well seated and resolution of the PVR confirmed on two-dimensional (Figure 5, Video 5) and three-dimensional (Figure 6, Video 6) TEE, with no left ventricular outflow obstruction. The mean mitral gradient by TEE was reduced from 7 to 4 mm Hg (Figure 2B).

The patient’s hemolytic anemia improved rapidly postprocedure, and they were discharged. Outpatient clinic review at 1 month postprocedure found the patient to be asymptomatic. Repeat TTE at 1, 5, and 10 months postprocedure showed a well-seated mitral ViViV, with no abnormal intravalvular regurgitation and only mildly elevated mean pressure gradients at 9, 10, and 8 mm Hg, respectively, believed to reflect the altered annular area from the procedure. Hemoglobin levels remained stable, and no further markers of hemolysis were recorded.

## Discussion

This case highlights the complexities of managing patients with histories of multiple valve replacements, especially when the risk of redo sternotomy is prohibitive. ViV procedures are gaining popularity as a safe and effective treatment in patients with degenerating bioprostheses.^2^ There are few published case reports of ViViV procedures. The indications for this treatment are likely to expand as safety and efficacy outcomes are demonstrated. Procedural efficacy approaches 90% for ViV implantation.^3^

Correct valve sizing in transseptal mitral ViV procedures is important to prevent future complications. Valve undersizing, as was the case with our patient’s initial emergency ViV procedure, is a cause of PVR and valvular hemolysis.^4^ Transcatheter heart valves are usually sized slightly larger than the internal diameter of the surgical valve being replaced, with deployment of valve struts in a conical shape, flaring in the ventricular aspect.^2^ Internal prosthesis diameters <22 mm are predictors of prosthesis-patient mismatch.^5^ Our patient’s initial 23-mm valve was undersized and also had markedly reduced valve area due to angulation from migration.

Valve migration results from the substantial systolic forces to which prostheses in the mitral position are exposed. Migration risk is increased by even slight valve displacement.^4^ Migration risk is therefore higher in circumstances in which the valve orifice is not completely circular, which may be the case with some rigid circular surgical valve rings, or unevenly distributed mitral annular calcification. Reports of mitral ViV migration, especially delayed migration, are rare in the literature. Bapat *et al.*^6^ reported two cases of delayed migration at 1 and 3 months, considerably earlier than the 12-month delay in our patient. The exact frequency of this complication is unknown but likely not dissimilar to the rates seen in transcatheter aortic ViV procedures at approximately 1%.

Preventing complications requires meticulous preprocedural planning and multimodality imaging. The inability to perform preoperative CCT resulted in relative undersizing and lack of ventricular flaring of anchoring valve struts during the previous ViV procedure. Real-time imaging modalities such as TEE are vital during transcatheter valve implantation to ensure optimal final valve placement, adequate leaflet movement, minimal valvular regurgitation, and reduction in transmitral gradients.^4^ Echocardiography modalities are also used in the diagnosis of ViV complications. Specifically, TTE is appropriate for monitoring ejection fraction, PVR, and gradients through the MV, which may also suggest migration or orifice interruption due to pannus, thrombus, or leaflet abnormality. Additional imaging is often required. Additionally, TEE allows the assessment of leaflet appearance, function, and motion and provides excellent imaging of the atrial side of the MV. Features of thrombus and alternative causes of emboli such as atrial or ventricular thrombi and infective endocarditis can often be differentiated. Three-dimensional TEE can be superior to assess vegetations, prosthetic rings, and prosthetic valve struts in patients with previous prostheses. A disadvantage of three-dimensional TEE is its lower temporal resolution (i.e., frame rate). Diagnostic TEE may require a broader viewing plane, especially to assess larger areas of PVR (Video 3). Higher frame rates may be required to assess finer valve detail such as small thrombi or tiny valvular leaks. This can be achieved by viewing only a narrow sector width (Video 4). Full-volume acquisition modes that capture data over multiple beats and display after stitching images together can improve spatial and temporal resolution. However, this requires an artifact-free electrocardiogram without arrhythmias for accurate image gating and the absence of patient and sonographer movement to prevent “stitching artifact,” in which misalignment between consecutive data captures results in a distorted final image. This method of image acquisition is more time consuming and is not always of diagnostic benefit. Imaging cardiologists are required to obtain high-quality images that maximize efficiency, especially during transcatheter procedures when patients are anesthetized or unstable.

There are no guidelines on recommended follow-up intervals for patients who have undergone ViV procedures in the mitral position. In a detailed review, Hensey *et al.*^7^ recommended that postprocedural TTE be performed at 1, 6, and 12 months, followed by annual TTE thereafter. Annual review with TTE is recommended by the American College of Cardiology for transcatheter replacements in the aortic position.^8^ The timing of follow-up echocardiography in our patient was brought forward given the rare complication following the first ViV implantation.

## Conclusion

This case illustrates the feasibility of using a transcatheter mitral ViViV procedure to address the rare complication of delayed migration of a ViV prosthesis resulting in significant intervalvular regurgitation and hemolytic anemia. Multimodality cardiovascular imaging is crucial to the diagnosis and procedural planning in patients with complex valvular issues, especially with regard to valve sizing. Future research on the optimum frequency of follow-up echocardiographic imaging and long-term outcomes in this patient population is required.

## Ethics Statement

The authors declare that the work described has been carried out in accordance with The Code of Ethics of the World Medical Association (Declaration of Helsinki) for experiments involving humans.

## Consent Statement

Complete written informed consent was obtained from the patient (or appropriate parent, guardian, or power of attorney) for the publication of this study and accompanying images.

## Funding Statement

The authors declare that this report did not receive any specific grant from funding agencies in the public, commercial, or not-for-profit sectors.

## Disclosure Statement

The authors report no conflict of interest.

## Supplementary Data

Video 1 Two-dimensional TEE, midesophageal long-axis (157°) view, demonstrates migration of mitral ViV prosthesis toward the left atrium (LA) with significant angulation.

Video 2 Two-dimensional TEE, midesophageal long-axis (155°) view, with color flow Doppler, demonstrates moderate PVR between 27-mm bioprosthetic MV and previously placed 23-mm transcatheter ViV implant.

Video 3 Three-dimensional TEE, volume-rendered reconstruction display with color flow Doppler, en face view from the perspective of the LA, demonstrates significant transvalvular and supraskirtal regurgitation between 9 o’clock and 1 o’clock positions during systole.

Video 4 Three-dimensional TEE, with color flow Doppler, volume-rendered reconstruction display, en face view from the perspective of the LA, demonstrates significant mitral transvalvular regurgitation (TVR).

Video 5 Two-dimensional TEE with color flow Doppler, midesophageal long-axis (80°) view, demonstrates resolution of the TVR following insertion of the 26-mm valve within the previous 23-mm transvalvular implant in the 27-mm bioprosthetic MV.

Video 6 Three-dimensional TEE, volume-rendered reconstruction with color flow Doppler display, en face view of the MV from the perspective of the LA, demonstrates resolution of TVR following insertion of 26-mm transvalvular implant over previous 23-mm transvalvular implant in 27-mm bioprosthetic MV.
